# Mothers and fathers with schizophrenia: Treatment and quality of life

**DOI:** 10.1192/j.eurpsy.2023.2313

**Published:** 2023-07-19

**Authors:** S. Perez-Sanchez, I. Martin Herrero

**Affiliations:** Psychiatry, Los Arcos del Mar Menor Universitary Hospital, murcia, Spain

## Abstract

**Introduction:**

Schizophrenia is a chronic disease that deteriorates the functionality of patients, especially when raising a family and caring for children. We are interested in analyzing the characteristics of mothers and fathers diagnosed with schizophrenia and their degree of global activity when switching from oral treatments to injectable treatments.

**Objectives:**

1 To evaluate the quality of life and functional level of mothers with schizophrenia treated with quarterly paliperidone palmitate. 2. To compare the quality of life and functional level when switching from oral treatment to long-acting injectables.

**Methods:**

Participants were 3 mothers and 3 fathers, 33-40 years old, with a diagnosis of schizophrenia in monotherapy who changed treatment with monthly paliperidone palmitate to quarterly paliperidone palmitate LD IM (525 mg/every 12 weeks). Retrospective data collection. QLS quality of life scale.

**Results:**

Six patients were included, caregivers of 1 child (80%), 2 children (20%) who met the inclusion criteria and completed the questionnaires. After its application and correction by means of non-parametric tests (N<30). During oral treatment, scores are observed in the QLS questionnaire of: intrapsychic functions mean 34.2, interpersonal relationships mean 19, instrumental role mean 8, daily activities mean 8. After 12 months of treatment with intramuscular paliperidone palmitate (injectable every 12 weeks ) scores were obtained: intrapsychic functions mean 36, interpersonal relationships mean 23, instrumental role mean 15, daily activities mean 11. A better functioning of the patients was observed in the instrumental categories and daily activities. As well as the patients referred better adherence to treatment.

**Conclusions:**

In our experience, injectable long-acting Paliperidone Palmitate every 12 weeks is associated with the perception of a better quality of life in parents with schizophrenia and increases administration facilities as well as planning in their daily lives.

**Disclosure of Interest:**

None Declared

